# Contribution of low-level motion to position shifts

**DOI:** 10.1167/jov.24.8.13

**Published:** 2024-08-23

**Authors:** Donald I. A. MacLeod, Patrick Cavanagh, Stuart Anstis

**Affiliations:** 1 Department of Psychology, University of California San Diego, La Jolla, CA, USA; 2 Department of Psychology, Glendon College, North York ON, Canada; 3 CVR, York University, North York ON, Canada; 4 Psychological and Brain Sciences, Dartmouth College, Hanover, NH, USA

**Keywords:** motion, motion-induced position shift

## Abstract

Motion can produce large changes in the apparent locations of briefly flashed tests presented on or near the motion. These motion-induced position shifts may have a variety of sources. They may be due to a frame effect where the moving pattern provides a frame of reference for the locations of events within it. The motion of the background may act through high-level mechanisms that track its explicit contours or the motion may act on position through the signals from low-level motion detectors. Here we isolate the contribution of low-level motion by eliminating explicit contours and trackable features. In this case, motion still supports a robust shift in probe locations with the shift being in the direction of the motion that follows the probe. Although robust, the magnitude of the shift in our first experiment is about 20% of the shift seen in a previous study with explicit frames and, in the second, about 45% of that found with explicit frames. Clearly, low-level motion alone can produce position shifts although the magnitude is much reduced compared to that seen when high-level mechanisms can contribute.

## Introduction

A moving background can shift the apparent separation between two flashed probes (Cavanagh & Anstis, 2013; Anstis & Cavanagh, 2017; Özkan, Anstis, ’t Hart, Wexler, & Cavanagh, 2021) so each probe is displaced in the direction of the motion after the probe flashed (Movie 1). On the left in Movie 1, the two probes are colored disks that are physically aligned, one above the other; however, perceptually they appear separated by about the distance the background frame has moved. On the right, the two probes are colored squares of identical size but the blue square appears about twice the size of the red, about one third the change of the background size (4:1). In these two cases, the moving background has three aspects that may affect the perceived locations or sizes of the flashed tests. First, the moving boundaries of the background establish a reference frame and locations of events within it may be defined relative to that frame. Second, the moving boundaries may engage high-level motion mechanisms that track their locations (Cavanagh, 1992). Third, the background frame has low-level motion that, itself, may shift apparent locations. Explicit boundaries have been shown to produce several motion-induced position effects (Nijhawan, 1994; Whitney & Cavanagh, 2000; Eagleman & Sejnowski, 2007; Cavanagh & Anstis, 2013). These effects may be driven by the low-level motion of the boundaries or by high-level tracking mechanisms.

**Movie 1 (Left).** (Left) In the frame effect (Özkan et al., 2021; Cavanagh et al, 2022), the outline square moves left and right while blue and red disks are flashed in alternation at each motion reversal. Although the discs are physically aligned, the blue directly above the red, they appear widely separated. (Right) In the Expansion Contraction effect (Anstis & Cavanagh, 2017), the banded background texture expands and contracts over a fourfold range. The red and blue outline squares flash in alternation at each motion reversal and although of identical size, the blue square appears about twice the size of the red. Movie is available on the journal website.

**Movie 1 (Right).** 

**Movie 2 (Left).** (Left) The random dot background moves left and right and the red disks flash at the motion reversals, one vertically above the other. Here the top disk flashes when the dot motion is at its rightmost extreme (1) and the bottom disk flashes when the dot motion is at its leftmost extreme (2). The top red disk appears shifted to the left of the bottom, in the direction of motion that follows the presentation of the top disk. (Right) The random dots now reverse contrast each time they move (step through the movie to see this) and they are perceived to move in the direction opposite to the physical displacement of the dots. As in the example above, the two red disks flash in alternation at each reversal but now the perceived tilt corresponds to the perceived, not physical direction of the random dots. The motion after the top flash is perceived to be rightward, and the top red disk is perceived to the right of the bottom disk. Movie is available on the journal website.

**Movie 2 (Right).** 

Several articles have examined the role of low-level and high-level motion in driving position shifts. High-level motion alone can support the full illusory displacement seen for test flashes in the frame effect when the frame is color or texture defined (Cavanagh et al., 2022). The motion-induced position shift is seen when the moving object is visible only through a slit that eliminates retinal motion (Watanabe, Nijhawan, & Shimojo, 2002). Low-level motion alone can also produce position offsets, for example, when viewing a motion aftereffect (Nishida & Johnston, 1999; Whitney, 2006) or an array of stationary Gabors that appears offset in the common motion direction of the set of Gabors that have varying internal directions (Scarfe & Johnston, 2010). To compare the low-level and high-level contributions to a motion-induced shift (the flash grab), Kohler, Cavanagh, and Tse (2015) presented a test flash near an oblique border of a moving diamond figure (Lorenceau & Shiffrar, 1992) where the border had oblique low-level motion but horizontal global motion. The result was a shift primarily in the low-level direction but with a smaller bias in the global direction.

Here, we will isolate the effects that low-level motion has on position by using random dot fields to remove the effects of explicit, trackable moving boundaries. We will look at two versions of this boundary-less, motion-induced shift: translation and expansion-contraction.

## Experiment 1: Translation

**Figure 1.**
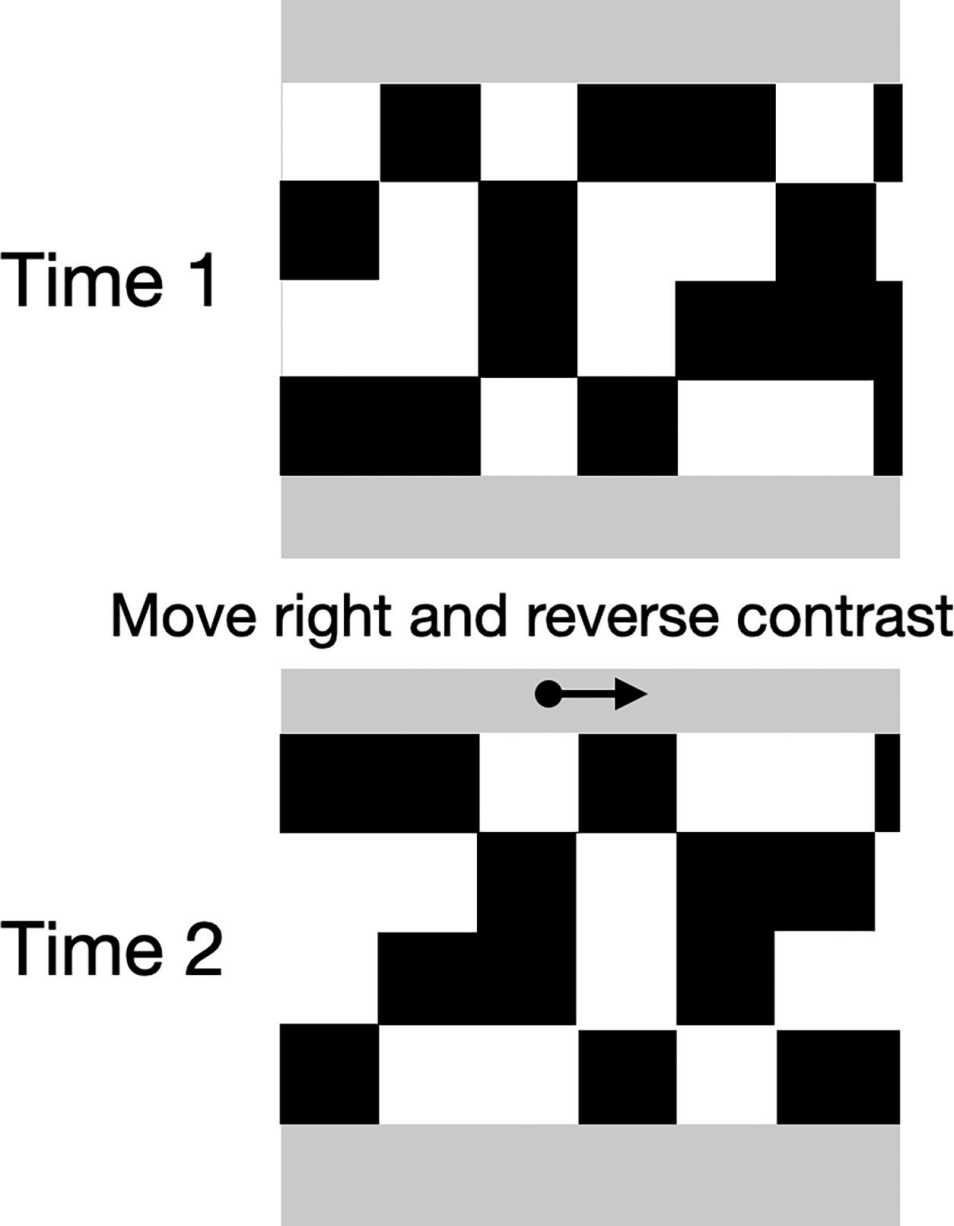
In reverse apparent motion, the dot pattern shifts, here to the right by one dot width, and reverses contrast. The perceived direction is opposite to the physical shift, to the left here (Anstis, 1970; Anstis & Rogers, 1975).

In this experiment, random dot fields drifted back and forth with test probes that flashed, one above the other, one at each reversal (Movie 2). The random dot field was bounded on the left and right by fixed edges so there was no boundary moving with the dots. In the first condition, a regular random dot field moved left and right providing motion but possibly also local feature cues. In the second condition, the dot field was in reverse apparent motion (alternating contrast on each frame, Anstis, 1970; Anstis & Rogers, 1975) so that its motion went in the direction opposite to its local features (Figure 1). This provides a stronger test of the effect of motion on position when no identifiable features are moving in the perceived direction.

### Methods

#### Participants

Eleven individuals, including three of the authors, participated remotely in this experiment (two women; age range: 25–86, mean 46). All participants were right-handed and had normal or corrected-to-normal vision, and, other than the three authors, all were naive to the purpose of this study. Informed consent was obtained from each participant before their experimental sessions as part of the recruitment email (see https://osf.io/rwv6s/). The procedures were approved by the Committee for the Protection of Human Subjects at Dartmouth College.

#### Apparatus

The experiment consisted of a set of movies presented to the participants in their web browser accessed at this URL https://cavlab.net/Demos/DMX. Each participant ran the study on different laptop or desktop computers. The monitor size, browser window size and viewing distance were not controlled. The participants reported that their screen sizes ranged from 11″ to 23″ and viewing distance varied from around 57 cm (typical arm's length) to 70 cm.

#### Procedure

**Figure 2.**
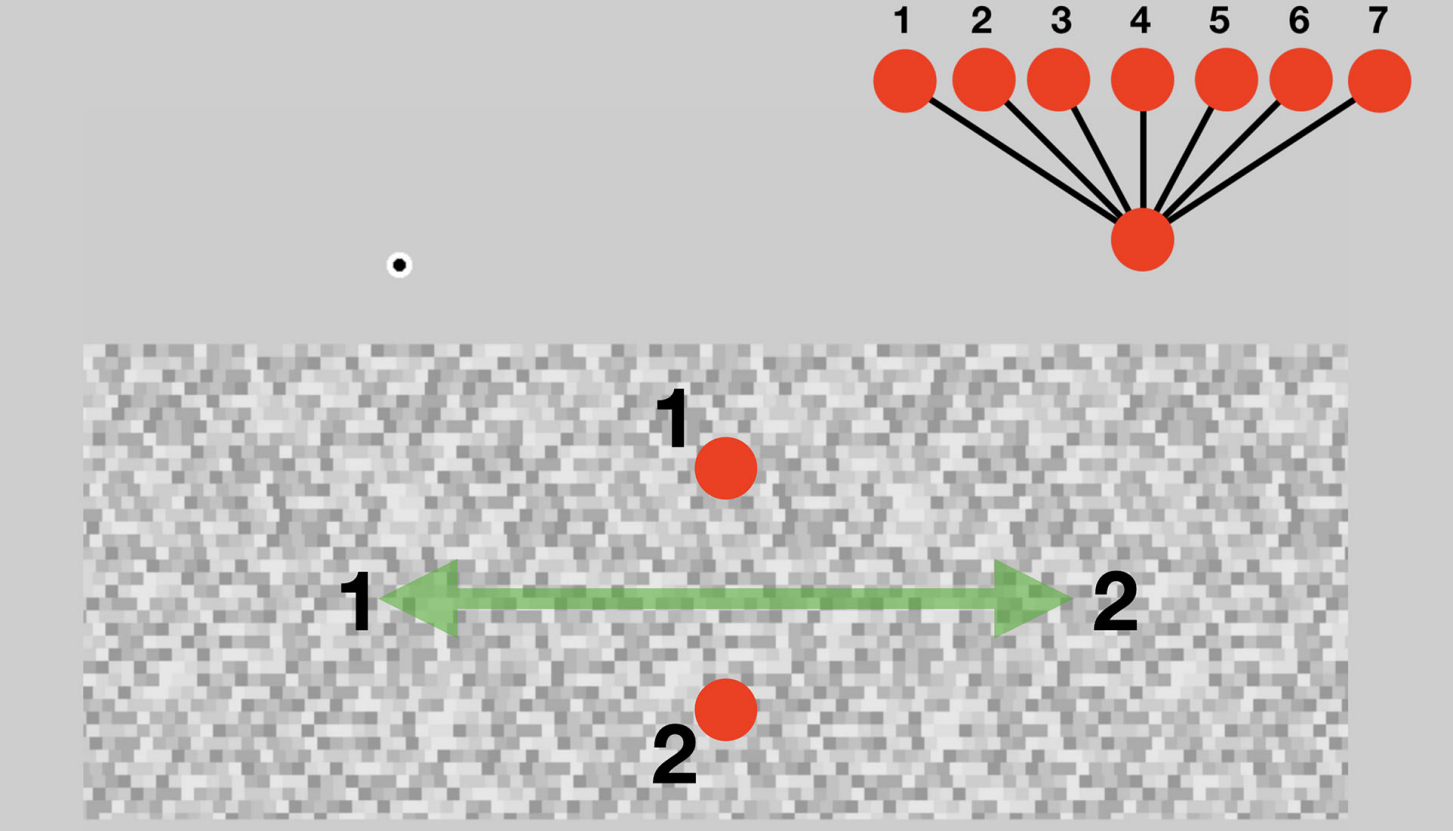
A typical stimulus display for Experiment 1. The random dot field moved left and right either in normal or reverse apparent motion, with the left and right borders static. In this condition, the top disk flashed when the dot motion was at its leftmost extreme (1) and the other flashed when the dot motion was at its rightmost extreme (2). Both disks were red and in one condition vertically aligned (as shown here). In two other conditions, the disks were offset relative to each other, either clockwise or counterclockwise. Participants judged the tilt seen between the two disks and matched it to one of the possible tilts shown above and to the left.in the display. The reversing motion continued until participants had made their choice and clicked to move to the next test. The experimental displays can be seen at https://cavlab.net/Demos/DMX.

The participants received a recruitment email that outlined the experiment and specified that only those who consented to participate could then load the experiment web pages and return their responses (sample recruitment email at https://osf.io/rwv6s/). Figure 2 shows the layout and logic of the experiment. The background motion covered 800 × 300 pixels of the 1024 × 768 pixels in the browser window. Participants may have rescaled their browser window but, if so, the size ratios remained the same. The disk sizes were 5% of the moving background's width (40 pixels diameter). The random dot background had dots that were 1% of the background's width (8 × 8 pixels) that each took randomly one of eight luminance values between the minimum and maximum, with 60% contrast between the minimum and maximum. The background moved repeatedly back and forth horizontally, reversing every 450 ms over a path (160 pixels) that was 20% of the width of the background dot field. The dots moved one direction for 333 ms in 10 steps, then paused for 117 ms while the probe disk flashed red for 67 ms in the middle of that pause, and the dots then moved in the opposite direction for another 333 ms, and paused again for 117 ms while the second probe flashed for 67 ms. The two probes were separated vertically by 100 pixels (12.5% of the background width) and had one of three physical offsets, either tilted to the left by 100 pixels, 12.5% of the background's width (67.5% of the background's path), vertically aligned, or tilted to the right by 100 pixels, 12.5% of the background's width. On half of the trials, the contrast of the random dots remained the same at each displacement of the background, in the other half, the contrast reversed on each step. Participants were instructed to fixate the dot above and to the left of the movie center and judge the apparent angle between the top and bottom probe and choose which tilt among the seven on the top right of the display best matched their perception (Figure 2), where intermediate values (e.g., 5.5) were allowed. The background continued to move back and forth with a probe flashing at each reversal until participants were ready to report their judgment. After recording their choice on the response sheet (see the recruitment email), they clicked on the current display to move to the next test. There were 12 tests (three probe offsets and two background motions, normal and reverse contrast, and one test with leftward motion after the top flash and one with rightward motion after the top flash) and the participants repeated the 12 tests four times. The experiment took about 15 minutes to complete. The participants emailed their response sheets to the experimenters. Stimuli, code, and results are available at https://osf.io/rwv6s/.

### Results

**Figure 3.**
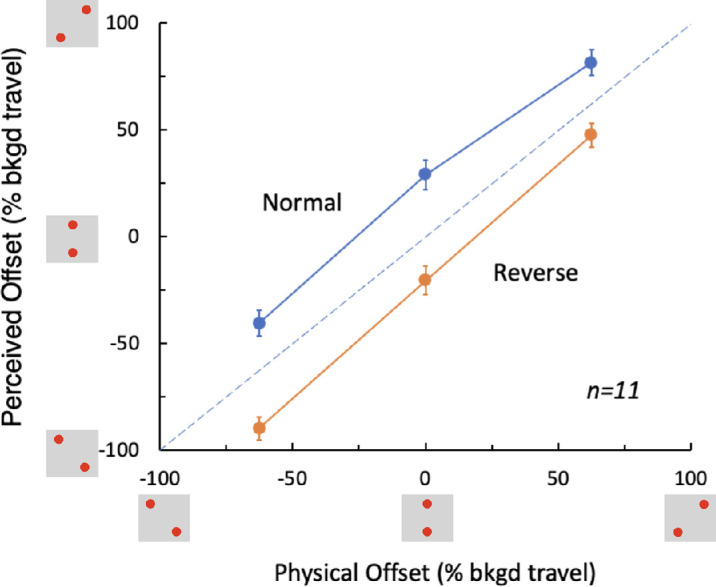
Perceived offset of flashed disks as a function of their physical offset, in percent of background travel. The two flashes could be tilted left or right or vertically aligned (0%) and the judgments of their actual tilt reveal an extra offset in the direction of the motion following each flash for the normal background motion (blue symbols). The perceived tilt was greater than the physical tilt (the three data points all lie above the dashed diagonal line) for all three values of physical tilt. On the other hand, for the background in reverse apparent motion (orange symbols), the offset was always less than the physical tilt, indicating a shift in the direction of the perceived as opposed to physical motion. The vertical axis shows positive values for clockwise tilt which corresponds to the half of the trials that had motion to the right following each flash of the upper disk. Data for the trials with leftward shifts following the flash of the top disk have been reversed before averaging to account for the opposite background motion. The vertical bars indicate ±1.0 SE.

Figure 3 shows the perceived locations of the probes corresponding to the reported tilts for the three physical probe offsets. Results from leftward motion trials and rightward motion trials are combined by reversing the offset's sign for the leftward trials and averaging. For the normal background motion, the perceived positions of the probes were offset in the direction of the physical motion that followed each flash (blue symbols, Figure 3). This offset was similar across the three physical positions of the flashes. This result was reversed for the background in reverse apparent motion (orange symbols, Figure 3) so that the perceived offsets were in the direction opposite to the displacement of the physical dot pattern. They were, however, in the same direction as the perceived motion following each flash.

**Figure 4.**
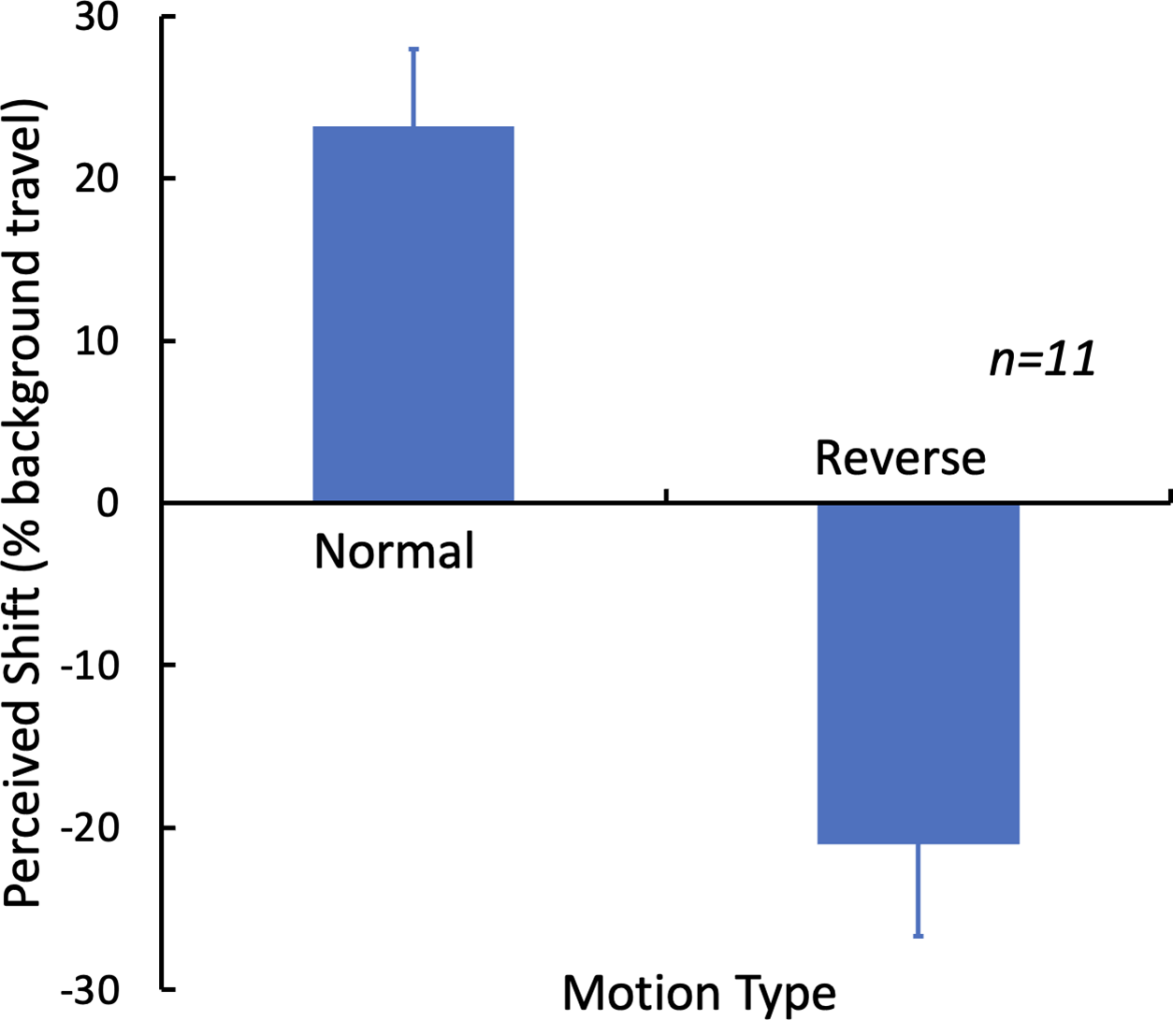
Perceived shift: the difference between the reported tilt and the physical tilt of the two probe disks, averaged across the three physical tilts. The error bars show 1.0 SE.

Figure 4 show the position shifts averaged across the three physical arrangements. The absolute values of these average position shifts did not differ significantly between the normal and reverse motion conditions. In both cases, the shift was about 20% of the background travel, but in opposite directions. The perceived shifts appear to be determined by the perceived direction of motion even in the absence of any trackable landmarks in the moving background.

### Conclusion

The moving field of dots generated a shift in perceived location that was about 20% of the distance travelled by the dots. This was true for the ordinary dot motion and equally true for the reverse apparent motion, although in the opposite direction. The similarity of the amplitude of the motion-induced shifts in both cases argues that the effects were based purely on the low-level motion signal and not on any cues of local clumps of dots that provided some reference frame. The reduced strength of the effect compared to the standard frame effect (about 100% of the distance traveled, Özkan et al., 2021) indicates that while the low-level motion can, on its own, contribute to a position shift, it is much less effective than the moving boundaries of the standard frame.

## Experiment 2: Expansion-contraction

In this experiment, we examine a flash grab version (Cavanagh & Anstis, 2013) of motion-induced position shift. We use an expanding and contracting stimulus (Anstis & Cavanagh, 2017) that has a massive effect on the perceived size of two identical squares presented at each reversal (Movie 1, right). The magnitude of the perceived size difference between the two squares in that study was about one third of the magnitude of the size change of the banded dot background. The stimulus has explicit boundaries in the textured background and that, rather than the dot motion, may have been the principal source of the illusory shifts. Here we remove those boundaries and test five versions of the stimulus (Movie 3): (A) a regular expansion and contraction of the random-dot field; (B) a limited-lifetime version that has the motion of the dot fields but eliminates any persisting local features that might provide a frame of reference; (C) a reverse apparent motion version that has the motion in the opposite direction to the size change of the dots; (D) a version where the dots grow and shrink in place, providing the looming and receding aspect of the expansion and contraction but without any net motion; and finally, (E) we now restore the explicit contours to mimic the original 2017 version and give an estimate of the extra contribution, if any, of the contours.

**Movie 3A.** (**A**) A background of regular random dots expands and contracts with green and magenta squares flashing alternately at each reversal. The squares have identical sizes. **(B**) Same as A but now the motion has a lifetime of two frames. (**C**) Same as A but now the contrast reverses on each frame. (**D**) The size of the random dot field increases and decreases but it is a different field of dots on each frame. (**E**) Same as A but now there is an additional set of explicit contours that increase and decrease in size along with the random dot background. Movie is available on the journal website.

**Movie 3B.** 

**Movie 3C.** 

**Movie 3D.** 

**Movie 3E.** 

### Methods

#### Participants

Four individuals, including one of the authors, participated in this experiment (two women; age range: 19-76, mean 44). Three participants were right-handed, and all had normal or corrected-to-normal vision. Other than the one author, they were naive to the purpose of this study. Written, informed consent was obtained from each participant before their experimental sessions. The study was approved by the Human Participants Review Sub-Committee of York University's Ethics Review Board.

#### Apparatus

All stimuli were generated on an Apple Macintosh G4 computer with custom software written in C using the Vision Shell Graphics Libraries (Comtois, 2003). The stimuli were presented on an AOC FreeSync 24″ LCD monitor using a setting of 800 × 600 pixel resolution. The test display was 500 × 500 pixels, covering 25° × 25° of visual angle at a 57 cm viewing distance, refreshed at 60 Hz. Response adjustments were made with a track pad.

#### Stimuli

The screen was filled with random light and dark dots at 50% contrast. The initial dot sizes were 0.1° × 0.1°, increasing to 0.4° × 0.4° pixels at the end of the expansion phase. The dot sizes increased by a fixed ratio of 14.87% on each of 10 steps in the expansion phase to reach the fourfold increase and decreased by the same ratio steps on the contraction phase. The duration of both the expansion and contraction phases was 450 ms and the motion pause at the end of each phase was 50 ms. The two flashed square outlines were alternately green and magenta presented for 50 ms, centered over the static background during each 50 ms pause. The reference green square was 7.5° × 7.5° with a contour width of 0.5° and it was always flashed on the smallest dot background prior to the expansion phase. The size of the adjustable magenta outline square was set by the participant using the trackpad and its contour width was also 0.5° pixels.

#### Procedure

Each trial began with a beep following which the expansion and contraction cycles were presented continuously with the green and magenta outline squares flashing at each motion reversal. There were five different background types randomly interleaved with 5 repetitions of each.

##### Regular motion

The dots expanded then contracted uniformly over the 10 steps of each phase (Movie 3A). Clumps of dots became relatively large at maximum expansion and may have offered trackable features.

##### Limited-lifetime motion

The dot patterns changed size on each step but on alternate steps, a new random dot pattern of the appropriate size was presented (Movie 3B). This created motion signals on half the steps (where the dot patterns were retained) and eliminated persisting feature clumps for tracking.

##### Contrast reversed motion

The dot patterns changed size on each step as in the regular motion case but now the patterns reversed contrast on each step as well (Movie 3C). This produces reverse apparent motion so the low-level motion for these stimuli is in the direction opposite to the physical expansion-contraction pattern.

##### Size-only

The dot patterns changed size appropriately on each step but they were different random patterns (Movie 3D). In this case, the sizes of the dots grow and then shrink to capture the looming and receding aspect of the regular motion, but now there is no local motion. This is the same as a limited lifetime stimulus with just one frame lifetime.

##### Banded motion

The dot patterns changed size as in the regular motion case but now a set of 4 nested outline squares expanded and contracted along with the dots (Movie 3E). This mimics the banded pattern used in the original (Anstis & Cavanagh, 2017) article but has four bands instead of the two of the original. The potential effect of the contour is assumed to drop off quickly with distance (Cavanagh & Anstis, 2013) and the reason for the additional bands is ensure at least one explicit contour is near the magenta square over the range of possible sizes it might take.

Using a track pad, participants adjusted the size of the magenta square until it appeared to match that of the reference green square. They were instructed to look around the display as they made their setting. When they were satisfied with their match, they pressed the space bar, and the next trial began. There were five conditions and five repetitions of each trial presented in random order for a total of 25 trials. The responses were self-paced, participants could take a break at any time. The experiment lasted about 15 minutes. The code, data, and analyses are available at https://osf.io/rwv6s/.

### Results

**Figure 5.**
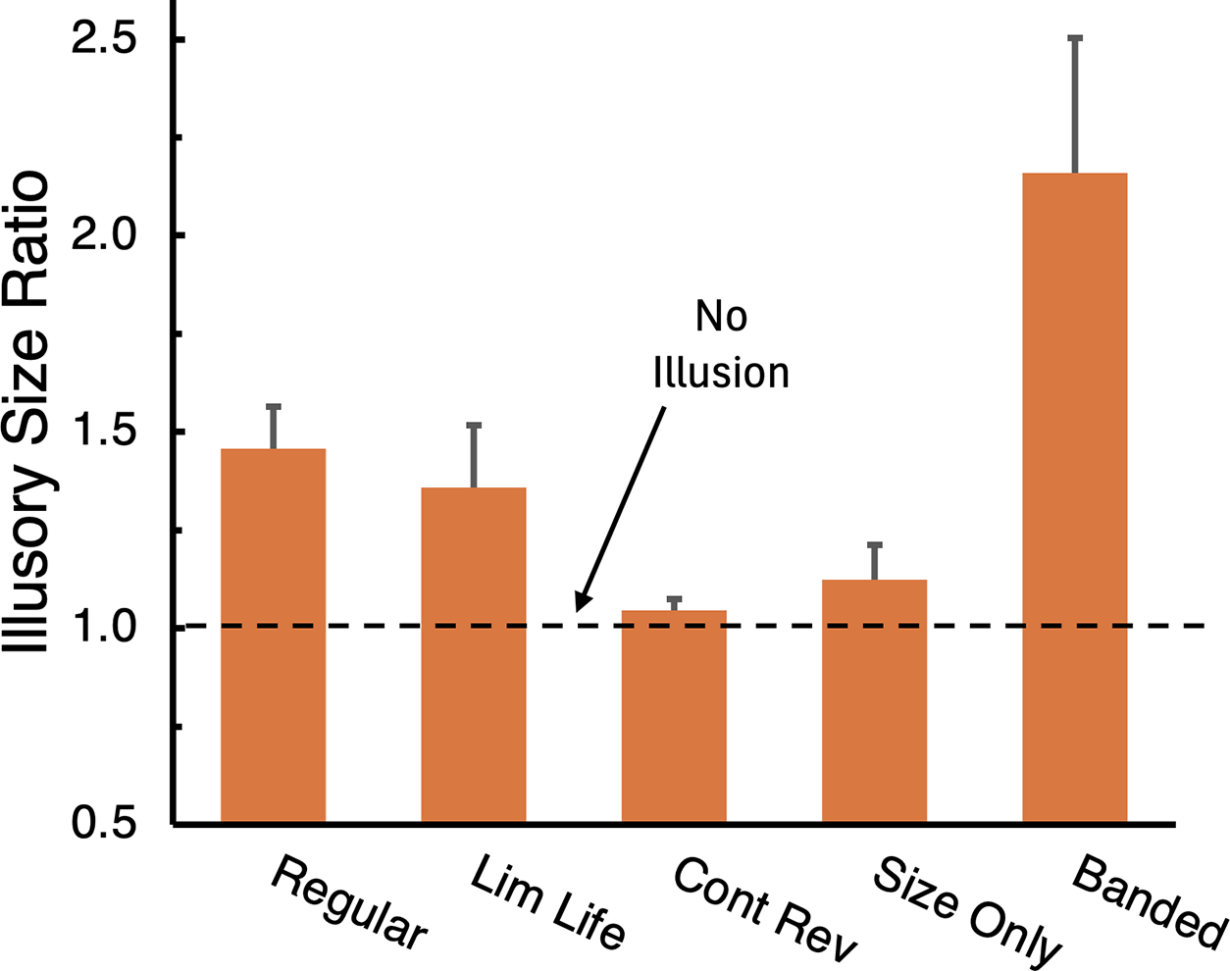
Illusory size ratios for the five motion conditions. A ratio of 1 indicates no illusion. The error bars show +1.0 SE.

The results are shown in Figure 5. With the Regular expanding and contracting background (Movie 3A), the magenta square looked smaller than the green square and had to be increased in size by 46% (leftmost bar, Figure 5) to match the apparent size of the green square. Each square's contours are shifted in the direction of the background motion that comes directly after it making the green square appear bigger and the magenta square appear smaller. Since the background undergoes a four-fold change in size (a 300% increase), the combined size shifts driven by the regular background motion is about one sixth of that. With the Limited Lifetime background motion (Movie 3B), there was less motion energy but also no persisting shapes in the random dots that could be followed. The result was slightly less effect: the magenta square needed only 36% increase in size to match the green square in size. With the Contrast Reversing motion, there was little or no apparent size difference between the green and magenta squares (Movie 3C). The reverse apparent motion suppressed the effect of the motion but did not reverse it as was the case in Experiment 1. In the Size Only condition (Movie 3D), the random patterns increased and decreased in size, but there was no local motion signals because the random pattern was different on each frame. The result again was little or no effect on the relative apparent sizes of the two test squares. Finally, with the reintroduction of the explicit Banded contours in the background (Movie 3E), the combined influence of the contours and background dot fields reinstated the large, more than 100% effect (a doubling in size) seen in the original article (Anstis & Cavanagh, 2017). This is one third of the physical size change of the stimulus, which quadrupled in size (4:1), a 300% increase.

### Conclusions

To isolate the motion effects here, we excluded two factors in the original expansion-contraction effect (Anstis & Cavanagh, 2017) that may strongly enhance the size change effects visible in Movie 1 (Right). First, the outer boundaries of the texture can be seen to expand and contract, which may provide a frame effect, generating impressions of size relative to the growing and shrinking frame. Second, the white areas in the backgrounds of Movie 1 (Right) are placed so that the contours of the target fall exactly on the background contours at the turnaround points. We found in an earlier study that the close alignment of the flashed probe with a contour greatly enhanced the position shift (Cavanagh & Anstis, 2013). In our new tests here, there was no global shape that expanded and contracted (except in the last version where they were re-introduced), and there were no explicit contours aligned with borders of the flashed squares. There was nevertheless a robust effect of the expanding and contracting dot fields on the perceived size of the flashed squares. This was maintained when the motion had limited lifetime to avoid any trackable clumps in the random-dot fields. When the motion signals were removed to leave only size changes or the motion signals were reversed, the size effects were no longer seen. The Banded version established that the presence of explicit contours near the flashed contours greatly enhanced the size effect, returning it to the magnitude found in the original study (Anstis & Cavanagh, 2017). We cannot say whether the increased effect is due to a stronger motion signal from the explicit contours or from the availability of trackable features that may engage high-level motion processes. Although the presence of the explicit contours did increase the illusory size differences, the contribution of low-level motion on its own is clear from the large effects in the two initial conditions with Regular random-dot motion and Limited Lifetime motion.

## Discussion

Several previous studies have shown that moving backgrounds can shift the perceived location of tests flashed near or on the background (Whitney & Cavanagh, 2000; Cavanagh & Anstis, 2013; Özkan et al., 2021; Cavanagh et al., 2022; Takao, Sarodo, Anstis, Watanabe, & Cavanagh, 2022; Adamian & Cavanagh, 2024). Although it is clear that the background's motion is the source of these position shifts, the effect may arise from either low-level or high-level motion mechanisms. Because these studies all used backgrounds with explicit contours, high-level mechanisms that track local features may be mediating the position shifts. Here we isolated the contribution of low-level motion by eliminating explicit contours and using random dot fields. In Experiment 1, we found that low-level motion alone produced strong position shifts in the direction of motion following the flash. The reverse apparent motion condition maintained that level of illusory position shift but in the perceived direction of motion, opposite to the displacement of any trackable features. Importantly, the strength of the displacement was about one-fifth the distance the background traveled. With explicit contours under similar conditions (Özkan et al., 2021), the perceived shifts can equal the distance traveled by the background frame. Clearly, low-level motion can drive a position shift, but it is not responsible for the large size of shift seen in other motion-induced position shifts.

The second experiment extended these findings using a contracting and expanding random-dot field that produces very large apparent change in size for briefly flashed test squares (Anstis & Cavanagh, 2017). Here we removed the explicit contours that were present in the original version and found again a robust effect. A limited lifetime version of the expanding and contracting dot field also showed a strong effect in the absence of any persisting shapes or clumps that might be tracked over time by high-level motion mechanisms. When the random dot field had only expansion and contraction of the dot sizes but no local motion signals (a different pattern on each frame), there was no size effect, indicating that it was the low-level motion signals, not size contrast that were driving the illusion. The anomalous case was the reverse movement in Movie 3C. Here the contrast reversed on every movie-frame, so that when the dots contracted or expanded, they produced a motion signal in the opposite direction. However, this had no effect on the size, indicating that something in the patterns might be tracked as they grew in size, offsetting the effect of the low-level motion signals in the opposite direction. The control stimulus with explicit contours showed the largest effect, consistent with the previously published results (Anstis & Cavanagh, 2017). Its effect was more than twice that of the low-level motion version (no explicit contours), again suggesting that although low-level motion does produce position shifts, the effects are much larger when high-level mechanisms can contribute.

Previous studies have shown that high-level motion is effective at producing position shifts (Watanabe et al., 2002; Scarfe & Johnston, 2010). Indeed, when a moving frame has little or no low-level motion energy (a second-order texture or equiluminous frame, Cavanagh et al., 2022), the large position shift for briefly flashed tests appears undiminished compared to that seen for a standard luminance-defined stimulus. In contrast, the evidence for an effect of low-level motion on perceived position is less direct. A motion aftereffect does produce shifts in apparent location (Whitney, 2006; Nishida & Johnston, 1999), and this effect would have to be attributed to low level-motion because the trackable features of the test are not physically moving. However, the effect size is relatively small as evidenced by the fact that it went unnoticed for more than a century before being directly measured. One study has compared the effect of low-level versus high-level motion (local vs. global) on the position shift (Kohler et al., 2015). Interestingly, they found that the low-level motion was more than twice as effective as the high-level, global motion in determining the perceived shift of the test flash.

In our two experiments, we eliminated the explicit contours and trackable features and still found a robust position shift, although at only a fraction of the magnitude seen when explicit contours are present. What mechanisms might be creating these position shifts driven by low-level motion? One could argue that the moving dot pattern provides a reference frame that influences the apparent locations of probes flashed on it. Even in the absence of identifiable landmarks, the dot pattern does have a speed and distance of travel that establishes a surface that appears to be in rigid motion despite its dynamic texture. The probes would then be seen in terms of their locations relative to this moving reference frame, as is proposed for the frame effect (Özkan et al., 2021), although with much less effect. Alternatively, because the flash and the motion reversals occur together, the probes may be bound to the background. The background motion would then be attributed to the flash (Cavanagh & Anstis, 2013), and this motion would shift the probe's perceived location. The mechanism of this shift may be extrapolation or position averaging as is often proposed for actually moving stimuli (e.g., Nijhawan, 1994; Krekelberg & Lappe, 2000). Further studies will be required to distinguish between these two alternatives but whatever the outcome, the results here show that low-level motion on its own is effective at shifting probe locations, but much less so than high-level motion.
